# Association between seaweed intake and risk of type 2 diabetes mellitus: a prospective cohort study

**DOI:** 10.1017/S0007114523002751

**Published:** 2024-04-14

**Authors:** Chaehyun Kim, Kyong Park

**Affiliations:** Department of Food and Nutrition, Yeungnam University, Gyeongbuk, 38541, Republic of Korea

**Keywords:** Seaweed, Type 2 diabetes mellitus, Cohort, Korea, Obesity

## Abstract

This study aimed to identify the longitudinal association between seaweed and type 2 diabetes mellitus (T2DM) in the Korean population. Data from 148 404 Korean adults aged 40 years and older without a history of T2DM, cardiovascular disease or cancer at baseline were obtained from the Korean Genome and Epidemiology Study data. The participants’ seaweed intake was obtained using a validated semi-quantitative food frequency questionnaire, and the diagnosis of T2DM was surveyed through a self-reported questionnaire during follow-up. The hazard ratio (HR) and 95 % confidence interval (CI) for T2DM were calculated using the Cox proportional hazard regression, and the dose–response relationship was analysed using a restricted cubic spline regression. Participants had a mean follow-up period of 5 years. Participants with the highest seaweed intake had a 7 % lower risk of T2DM compared with the group with the lowest intake (95 % CI (0·87, 0·99)). Interestingly, this association was stronger in those with normal weight (HR: 0·88, 95 % CI (0·81, 0·95)), while no association was observed in participants with obesity. Spline regression revealed an inverse linear relationship between seaweed intake and T2DM risk in participants with normal weight, showing a trend where increased seaweed intake is related to lower instances of T2DM (*P*_for nonlinearity_ = 0·48). Seaweed intake is inversely associated with the onset of T2DM in Korean adults with normal weight.

Seaweed refers to photosynthetic marine algae, and it can be classified into green (e.g. *Enteromorpha compressa*, *Codium fragile*), red (e.g. Porphyra, *Gelidium amansii*) and brown (e.g. *Saccharina japonica*, *Undaria pinnatifida* and *Sargassum fusiform*), depending on colour^(1,2)^. Seaweed is a rich source of both water-soluble and insoluble fibres^(3)^, which delay gastric emptying and thus lower postprandial spikes and enhance insulin sensitivity^(4,5)^. It has a low energy density and contains starchy carbohydrates^(6)^, thereby helping to lower the body mass index (BMI) and reduce insulin secretion^(7)^. Seaweed is also an excellent source of iodine, which helps improve fasting blood glucose levels^(8)^, and its antioxidants (e.g. vitamins B, C and E) eliminate reactive oxygen species, both of these benefits are conducive to managing type 2 diabetes mellitus (T2DM)^(9–12)^.

Most previous studies on the association of seaweed and its relevant supplement intake on the blood glucose levels were clinical trials^(13–15)^. According to a recent meta-analysis of eighteen clinical trials that investigated the association between brown algae (and its extract) and postprandial blood glucose and fasting glucose levels, intake of brown algae and its extract significantly lowered postprandial plasma glucose and fasting glucose levels^(15)^. Subsequent clinical trials reported consistent results. In a randomised three-way blinded crossover meal trial that analysed the associations between brown algae extracts (laminaria digitate and undaria pinnatiﬁda extracts) and blood glucose levels in twenty healthy adults aged 20–50 years in Denmark, participants who weighed less than 63 kg showed significantly lower glucose response than those in the control group 40–90 min after a meal containing laminaria digitata and undaria pinnatiﬁda^(14)^. In addition, participants who weighed 63 kg or more had significantly lower blood glucose response than the control group 120 min after a meal containing undaria pinnatiﬁda^(14)^. According to a 12-week double-blind randomised placebo-controlled trial examining sixty-four participants with overweight or obesity aged 18 years or older in Australia, the intervention group that consumed 4 g of seaweed extracts had a lower insulin response than the control group in an oral glucose tolerance test performed 2 h after intake of 75 g of glucose^(13)^.

Several studies were conducted in Japan to investigate the association between seaweed intake and metabolic disorders^(16–18)^. In the Japan Public Health Center-based prospective study on 40 707 men and 45 406 women aged 40–69 years using data from 1990–2009 to 1993–2012, both men and women with the highest seaweed intake had significantly lower risk of ischaemic heart disease than the lowest seaweed intake group^(16)^. In the Japan Collaborative Cohort Study for Evaluation of Cancer Risk on 40 234 men and 55 981 women aged 40–79 years using data from 1988 to 2009, women who consumed seaweed almost every day had significantly lower risk of all types of stroke and all types of cardiovascular disease (CVD) than the group that rarely consumed seaweed^(18)^. Furthermore, in the Circulatory Risk in Communities Study on 2792 men and 3377 women aged 40–79 years that analysed the association between seaweed intake and CVD using data from 1984 to 2000, the risk of all types of stroke and cerebral infarction was significantly lower among men who consumed the most seaweed than among those who did not consume seaweed^(17)^.

However, limited studies were conducted in some subgroups of the Korean population, and few studies have investigated prospective associations. In a cross-sectional study that analysed the association between seaweed intake and the prevalence of T2DM in 3405 Korean men and women aged 20–65 years using data from the 2005 Korea National Health and Nutrition Examination Survey, the prevalence of T2DM was significantly lower among men with the highest seaweed intake than among men with the lowest seaweed intake^(19)^. In a study analysing the association between seaweed intake and metabolic syndrome in 2588 pre-menopausal women aged 40–69 years using data from the 2005 to 2013 Multi-Rural Communities Cohort, the highest seaweed intake group had significantly lower risk for metabolic syndrome than the lowest seaweed intake group^(20)^.

In the participants with obesity, adipose tissues shift from an anti-inflammatory state to proinflammatory state and therefore induce systemic inflammation. This leads to endothelial dysfunction, metabolic disorders and peripheral insulin resistance^(21,22)^. Thus, we hypothesised that obesity, a known risk factor for T2DM, may influence the association between seaweed intake and T2DM risk.

In this context, this study aims to prospectively analyse the association between seaweed intake and T2DM risk in Korean adults aged 40 years and older and examine whether this association is moderated by obesity using Korean Genome and Epidemiology Study (KoGES)-Health Examinee Study (HEXA) data.

## Methods

### Data and participants

We used the KoGES-HEXA cohort data established primarily around twenty-six healthcare institutions in fourteen cities nationwide of Korea^(23)^. A detailed description of the cohort, along with a flow diagram of the baseline recruitment and follow-up processes, has been published previously^(24)^. The HEXA cohort comprised men and women aged 40 years and over who visited a health examination centre in an urban region to investigate the genetic and environmental factors of common chronic diseases in the Korean population. A baseline survey was administered to 173 200 participants in 2004, sampled via purposive/judgemental sampling and the first follow-up survey was conducted in 2012^(23)^. Participants’ demographic information, lifestyle factors, disease history, diet and psychosocial factors were collected using a questionnaire, and anthropometric measurements, body composition data and clinical test results were collected through baseline and follow-up examinations at baseline and follow-up. All survey procedures were conducted by trained interviewers using standardised protocols^(24)^.

Of 173 200 participants who completed the baseline survey, the following were excluded: participants with a total daily energy intake of 500 kcal or lower or above 5000 kcal (*n* 3689)^(25)^; participants diagnosed with T2DM, CVD or cancer at the baseline survey (*n* 20 931) and participants with missing seaweed intake data (*n* 176). As a result, 148 404 participants were included in our analysis. This study was approved by the Institutional Review Boards at the Korea Disease Control and Prevention Agency (IRB number: KU-IRB-15-EX-256-A-1) and Yeungnam University (IRB number: 7 002 016-E-2016-003). Data collection and analysis were conducted after all participants had provided written informed consent for use of their baseline data and biospecimens^(26)^.

### Demographic and lifestyle information

Demographic information, such as age and sex and lifestyle factors, such as smoking, drinking and physical activity, were obtained using a self-reported questionnaire included in the baseline survey^(23)^. BMI was calculated by dividing body weight (kg) by height squared (m^2^) and was classified as underweight (<18·5 kg/m^2^), normal (18·5–22·9 kg/m^2^), overweight (23–24·9 kg/m^2^) and obesity (≥25 kg/m^2^) according to the WHO obesity criteria for Asia-Pacific populations^(27)^. Subsequently, the participants were re-classified into two groups, those with normal weight (<25 kg/m^2^) and those with obesity (≥25 kg/m^2^). Smoking status was initially divided into ‘current smokers’, ‘past smokers’ and ‘non-smokers’ but was reclassified into ‘current smokers’ and ‘non-smokers’ based on the current smoking status. Alcohol consumption was initially divided into ‘current drinkers’, ‘past drinkers’ and ‘non-drinkers’ but was reclassified into ‘current drinkers’ and ‘non-drinkers’ based on the current alcohol consumption status. Physical activity was initially divided into ‘not active’, ‘lightly active’, ‘active’ and ‘very active’ but was reclassified into ‘low’, ‘medium’ and ‘high’ by merging ‘not active’ and ‘lightly active’ into one group.

### Dietary information

In this study, long-term nutrient intake was assessed using the semi-quantitative food frequency questionnaire (SQFFQ), developed and validated by the KoGES^(28)^. The list of foods in the SQFFQ included foods most frequently consumed by individuals aged 40–69 years based on 1998 Korea National Health and Nutrition Examination Survey data^(29)^. The first version of the SQFFQ containing 103 items was developed in 2001, and a revised version with 106 items was developed in 2004 after adding variables for seasonal foods, updating photos of foods to help accurately measure intake and revising recipes. The SQFFQ assesses the average intake frequency in the past year using a nine-level response (rarely ate in the past year, once a month, 2–3 times a month, 1–2 times a week, 3–4 times a week, 5–6 times a week, once a day, twice a day and 3 times a day) and average intake amount using a three-level response (0·5 servings, 1 serving and 1·5–2 servings). The average daily intake of twenty-three nutrients (e.g. energy) was computed using DS 24^(28)^ and the seventh edition food composition table of Korea^(30)^.

In the present study, we used the average values of food and nutrient intake reported in the baseline and follow-up surveys. Seaweed intake was calculated by adding the average intake of ‘laver’ and ‘kelp/sea mustard’. A single serving of ‘laver’ was 2 g, equivalent to about 1 large sheet of laver (8 cut pieces), and a single serving of ‘kelp/sea mustard’ was 5 g, equivalent to about 1 soup bowl. To assess total diet quality, Dietary Approaches to Stop Hypertension (DASH) diet score was calculated using the whole grains, vegetables, fruits, nuts and legumes, dairy products, sugar-sweetened beverages, meats and Na components^(31)^. Each of the eight components was rated on a scale of 1–5, and the scores of all component scores were summed; the healthy food components of whole grains, vegetables, fruits, nuts and legumes and dairy products gave high scores to the group with high intakes; conversely, unhealthy food components such as sugar-sweetened beverages, meats and Na were scored on a reverse scale^(31)^.

### Type 2 diabetes mellitus diagnostic criteria

For T2DM diagnosis, participants were asked to complete a questionnaire^(26)^. In baseline, T2DM was defined as diagnosis of T2DM by a physician at a hospital or clinic (including public health clinics), excluding self-diagnosis or diagnosis made by a doctor of Korean medicine/oriental medical doctor, pharmacist or nurse^(26)^. During the follow-up period, T2DM was defined as diagnosis of T2DM by a physician, fasting blood sugar ≥126 mg/dl or HbA1c ≥6·5 %^(32)^.

### Statistical analysis

This study used 2004–2016 KoGES-HEXA cohort data. The follow-up period was determined as the time interval between: (1) the date of the baseline survey and the date of T2DM diagnosis or (2) the date of the baseline survey and the date of the last-known-alive if T2DM was not diagnosed. For demographic factors, categorical variables were presented as frequencies (%), and continuous variables were presented as the mean ± SE. Association between seaweed intake and T2DM incidence was analysed using Cox proportional hazards regression analysis, and the results were presented as hazard ratios with 95 % confidence interval (CI). The *P*_for trend_ was calculated by applying the median value of each quartiles as a continuous variable. Potential confounders were identified through preliminary analysis and literature review^(33–36)^, and three statistical models were built. Model 1: unadjusted; model 2: adjusted for age and sex; model 3: additionally adjusted for smoking, drinking, physical activity, Dietary Approaches to Stop Hypertension (DASH) diet score and dietary intakes total energy. Interaction analyses and subgroup testing were conducted during the data analysis phase, not pre-specified. We investigated the combined effects of seaweed intake, demographic characteristics, lifestyle factors and other dietary elements on T2DM risk using multiplicative terms in our statistical models. Specifically, we considered age as both a continuous variable and in categories (below 65, and 65 and above), sex (men and women), obesity status (<25 kg/m^2^ and ≥ 25 kg/m^2^), smoking status (current smokers and non-smokers), alcohol consumption (current drinkers and non-drinkers), physical activity levels (analysed continuously and categorically as low and high), DASH diet scores (analysed continuously and categorically as low and high) and total energy intake (also treated continuously and categorised into low and high). For physical activity, DASH diet score and total energy intake, we divided into two groups of low and high based on median values.

The dose–response association between seaweed intake and T2DM incidence was analysed using a restricted cubic spline regression with three random knots after adjusting for the covariates used in model 3. All statistical analyses were performed using the SAS version 9.4 (Statistical Analysis System Institute Inc.), and statistical significance was set at *α* = 0·05.

## Results

After excluding 24 796 ineligible participants, data from 148 404 participants were analysed. The mean follow-up period in the participants was 5 years (279 293 person-years), and the incidences of T2DM per 100 person-year was 7·66.

### Participants’ baseline characteristics according to seaweed intake quartiles

Participants’ general characteristics according to seaweed intake quartiles are presented in Table 1. The mean age was approximately 52 years. The percentages of male participants in quartiles 1, 2, 3 and 4 were 38·6 %, 35·9 %, 31·6 % and 26·7 %, respectively, and the percentages of participants with normal weight was 68·5 %, 68·2 %, 68·7 % and 67·4 %, respectively. The percentages of non-smokers were 85·4 %, 86·6 %, 88·5 % and 89·5 %, respectively, and those of non-drinkers were 51·1 %, 51·7 %, 54·3 % and 57·3 %, respectively. The percentages of individuals engaging in high physical activity were 12·9 %, 12·8 %, 14·0 % and 13·4 %, respectively, and the mean DASH diet scores were 22·4, 23·6, 24·4 and 25·0, respectively. Total energy intake was 1547 kcal/d to 1954 kcal/d.

**Table 1.** Baseline characteristics according to seaweed quartile

	Quartile of seaweed intakes							
	Q1		Q2		Q3		Q4	
No. of participants	37 086		37 085		36 990		37 243	
	Median	Range	Median	Range	Median	Range	Median	Range
Seaweed (servings/week)	0·87	0·00–1·69	2·28	1·70–3·01	4·17	3·02–5·82	8·25	5·83–63·0
	Mean	SE	Mean	SE	Mean	SE	Mean	SE
Age (years)	52·4	0·04	52·3	0·04	52·5	0·04	52·6	0·04
	*n*	%	*n*	%	*n*	%	*n*	%
Sex								
Men	14 311	38·6	13 306	35·9	11 674	31·6	9940	26·7
Women	22 775	61·4	23 779	64·1	25 316	68·4	27 303	73·3
Obesity status*								
Normal weight	25 276	68·5	25 225	68·2	25 327	68·7	25 008	67·4
Obesity	11 636	31·5	11 751	31·8	11 550	31·3	12 098	32·6
Smoking status								
Non-smokers	31 554	85·4	31 967	86·6	32 605	88·5	33 179	89·5
Current smokers	5390	14·6	4961	13·4	4243	11·5	3895	10·5
Alcohol consumption								
Non-drinkers	18 870	51·1	19 106	51·7	20 022	54·3	21 249	57·3
Current drinkers	18 082	48·9	17 841	48·3	16 830	45·7	15 843	42·7
Physical activity								
Low	4827	22·6	4818	19·7	4442	18·4	3894	17·5
Mid	13 756	64·5	16 481	67·5	16 377	67·6	15 354	69·1
High	2753	12·9	3135	12·8	3400	14·0	2966	13·4
	Mean	SE	Mean	SE	Mean	SE	Mean	SE
Dietary approaches to stop hypertension diet score	22·4	0·02	23·6	0·02	24·4	0·02	25·0	0·02
Total energy intake (kcal/d)	1547	2·38	1678	2·33	1745	2·40	1954	2·99

Q, quartile.

* Obesity was defined as having a BMI ≥25 kg/m^2^ according to the WHO obesity criteria for Asia-Pacific populations.

Values are mean ± SE or as *n* (%).

### Association between seaweed intake and type 2 diabetes mellitus risk

Table 2 shows the hazard ratio (HR) and 95 % CI for the risk of T2DM according to seaweed intake among Korean adults. In all statistical models, higher intake levels of seaweed were associated with lower risk of T2DM (*P*_for trend_ <0·05). In the fully adjusted model including age, sex, smoking, alcohol consumption, physical activity, DASH diet score, and total energy intake, the highest seaweed intake group (Q4) had a 7 % lower risk of T2DM than the lowest seaweed intake group (Q1) (HR: 0·93, 95 % CI (0·87, 0·99), *P*_for trend_ = 0·01).

**Table 2. tbl2:** Hazard ratio (95 % confidence intervals) for risk of type 2 diabetes mellitus according to quartile of the seaweed intakes

	Quartile of seaweed intakes								*P*_for trend_
	Q1		Q2		Q3		Q4		
No. of participants	37 086		37 085		36 990		37 243		
No. of cases (%)	5000	13·5	5522	14·9	5856	15·8	5008	13·5	
			HR	95 % CI	HR	95 % CI	HR	95 % CI	
Model 1*	Ref		1·04	0·99, 1·10	1·02	0·97, 1·08	0·91	0·86, 0·96	<0·001
Model 2†	Ref		1·06	1·00, 1·11	1·05	1·00, 1·11	0·94	0·89, 1·00	0·002
Model 3‡	Ref		1·01	0·95, 1·07	1·01	0·96, 1·07	0·93	0·87, 0·99	0·01

Q, quartile; Ref, reference; HR, hazard ratio; CI, confidence interval.

* Model 1: unadjusted.

† Model 2: adjusted for age (continuous) and sex (men and women).

‡ Model 3: additionally adjusted for smoking status (current smoking and non-smoking), alcohol consumption (current drinking and non-drinking), physical activity (low, mid and high), Dietary Approaches to Stop Hypertension diet score (continuous) and total energy intake (continuous).

### Dose-dependent association between seaweed intake and type 2 diabetes mellitus risk

**Fig. 1.**
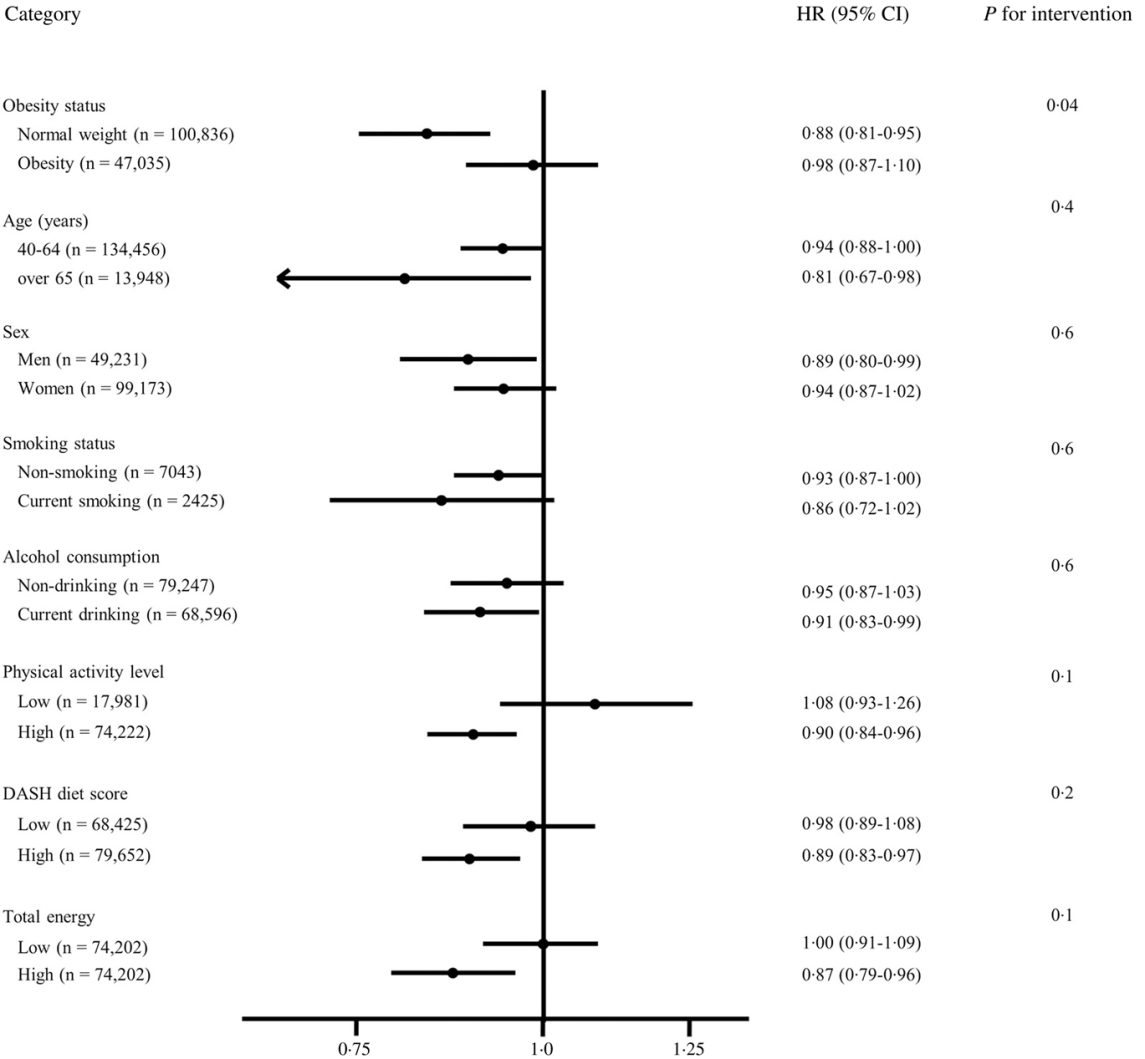
Effect of various demographic factors on the associations between seaweed intake and type 2 diabetes mellitus. The hazard ratios (HR) and 95 % CI presented were estimated using a Cox proportional hazards regression model, adjusted for all covariates listed in model 3 of Table 2. Additionally, for every individual subgroup, the reported outcomes are representative of the hazard ratio comparing quartile 4 to quartile 1. For physical activity, Dietary Approaches to Stop Hypertension (DASH) diet score and total energy intake, we divided into two groups based on median values.

Through interaction analyses and subgroup testing, we identified obesity status as a significant effect modifier in the association between seaweed intake and T2DM incidence. Thus, we presented the results of the association between seaweed intake and T2DM risk separately for obese and normal weight groups. Fig. 1 displays the spline curves showing HR and 95 % CI for a dose-dependent relationship between seaweed intake and T2DM risk. Age, sex, smoking, drinking, physical activity, DASH diet score and total energy intake were adjusted as covariates. The reference value for seaweed intake was 0·81 servings/week for both the obesity and normal weight groups. The nonlinearity between seaweed intake and T2DM was analysed for both groups, and the relationship between seaweed intake and T2DM risk in the normal weight group was dose dependent, with an inverse pattern observed as seaweed intake increased (*P*_for nonlinearity_ = 0·48).

### Subgroup analysis between seaweed intake and type 2 diabetes mellitus risk

**Fig. 2.**
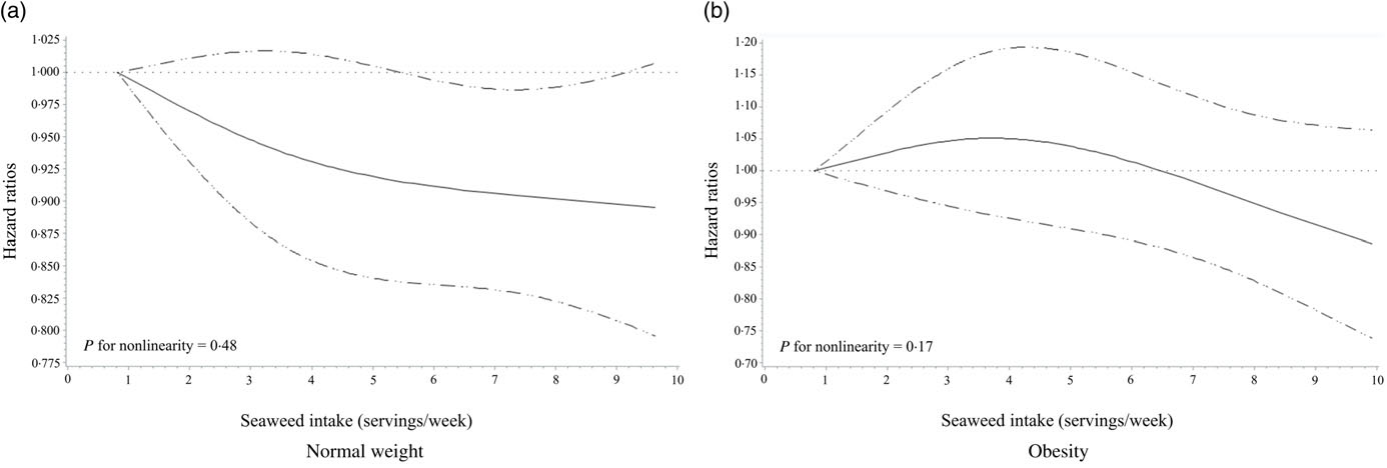
[Table tbl2]Hazard ratios (95 % CI) for the non-linear relationship between seaweed intake and type 2 diabetes mellitus in participants with (a) normal weight and (b) obesity, evaluated using restricted cubic splines. The model was adjusted for age, sex, smoking status, alcohol consumption, physical activity, Dietary Approaches to Stop Hypertension (DASH) diet score and total energy intake. Obesity was defined as having a BMI ≥25 kg/m^2^, according to the WHO obesity criteria for Asia-Pacific populations.

The association between seaweed intake and T2DM risk was further analysed by stratifying multiple demographic factors such as age, sex, obesity status, alcohol consumption, smoking status, physical activity, total energy and DASH diet score (Fig. 2). The relationship between seaweed intake and incident T2DM appeared to vary by obesity status. In the group of participants with obesity, an increase in seaweed intake did not correspond to a noticeable change in T2DM risk. Conversely, in the normal weight group, higher seaweed intake was associated with a lower occurrence of T2DM. However, the association between seaweed intake and T2DM risk was not affected by age, sex, alcohol consumption, smoking status, physical activity level, DASH diet score and total energy intake (all *P*_for interaction_ >0·05). After excluding 1154 participants whose seaweed intake was over 22·5 servings/week (the top 1 % of the distribution), the significant association remained identical (data not shown).

## Discussion

This study prospectively analysed the association between seaweed intake and T2DM risk in Korean adults aged 40 years and older who participated in the KoGES-HEXA study. Our findings indicated a significant inverse association between seaweed intake and the incidence of T2DM among participants with normal weight. However, this association was not observed in the group of participants with obesity. Further, this association appeared in a dose–response manner, but only in the group of participants with normal weight.

Although it varies by species, approximately 35–70 % of the dry weight of seaweed is dietary fibre^(37,38)^. Dietary fibre increases satiety, thus leading to decreased total energy intake and blood glucose levels^(39,40)^. Particularly, water-soluble fibre forms a viscous gel matrix, which delays gastric emptying and slows nutrient absorption in the small intestine, thereby lowering the chances of blood sugar spikes, which can occur when eating rapidly digested carbohydrates, such as white bread and refined sugar^(39)^. Moreover, seaweed has low energy density and starch content and can thus decrease the glycaemic load^(6,41)^ and uronic acid – the active ingredient of insoluble fibre — is known to lower blood glucose concentration owing to a low glycemic index^(42)^. Vitamins B, C and E, which are abundant in seaweed, have anti-inflammatory and antioxidant properties^(6,43,44)^, and polyphenols, including flavonoid, catechin, lignan and phenolic acid, have cytoprotective effects against oxidative stress; they reduce tissue injury by activating endogenous antioxidant defense mechanisms^(45)^. Fibre content in seaweed, such as fucoidan, laminin, alginin, ulvan, porphyran, polyphenols and peptides, have positive effects on gut microbiota^(46–48)^. Gut microbiota regulate immune markers, interact with food constituents and effectively regulate glucose and lipid metabolism, insulin sensitivity, energy balance and intestinal permeability. Gut microbiota have also been linked to a reduced incidence of T2DM^(49–51)^. The iodine in seaweed regulates thyroid functions and is involved in normal regulation of energy metabolism and blood glucose concentration^(52–54)^. Excessive Na intake or a high dietary Na to potassium ratio can have an adverse impact on T2DM risk by increasing fasting glucose and HbA1c levels^(33)^, and seaweed contains a low Na to potassium ratio between 0·1 and 1·5^(55)^. Moreover, the dieckol found in seaweed can improve glycaemia and insulin resistance following glucose load^(56)^; it also has antioxidant properties, through which it reduces oxidative stress injury induced by reactive oxygen species and nitrogen oxide caused by hyperglycaemia^(57)^. Finally, the phytosterol in seaweed decreases blood glucose concentration and has antioxidant, anti-inflammatory, anti-obesity and anti-diabetic effects^(58)^. In essence, seaweed intake is speculated to have positive effects on T2DM risk by reducing blood glucose elevation and insulin resistance and producing antioxidant and anti-inflammatory effects^(40,56,58)^.

In the present study, the state of obesity influenced the relationship between seaweed intake and T2DM onset, indicating obesity is a significant effect modifier. We observed a significant inverse association between seaweed intake and T2DM risk in individuals with normal weight, but this relationship was not shown in individuals with obesity. This association may be attributable to the interactions among seaweed intake, obesity, inflammation and T2DM. In an obese state, adipocytes release proinflammatory cytokines such as tumour necrosis factor-*α*, IL-6, IL-1*β* and chemokine and high-sensitivity C-reactive protein and prolonged inflammation results in cellular dysfunctions, endothelial dysfunctions and increased insulin resistance^(59,60)^. Furthermore, tissue dysfunctions result in excess release of free fatty acids, reactive oxygen species and inflammatory cytokines, which in turn can lead to cellular dysfunction, systemic dysfunction and cellular apoptosis^(61)^. Cellular and systemic dysfunction causes an overproduction of proinflammatory molecules and reactive oxygen species, impairs insulin sensitivity and glucose homoeostasis and brings complications^(61–63)^. Endothelial dysfunctions can manifest as hyperinsulinaemia by inducing excessive secretion of insulin by pancreatic *β* cells^(61–63)^. Therefore, we speculated that the lack of association between seaweed intake and T2DM in individuals with obesity may be due to functional and metabolic disorders caused by systemic inflammation, diluting the positive health effects of seaweed intake on T2DM risk.

With reference to an iodine content of 76 µg and 142·2 µg in one serving of ‘laver’ and ‘kelp/sea mustard’, respectively^(20)^, the median iodine intake from seaweed among our participants was 327·3 µg, equivalent to 2·18 times the recommended dietary allowance of iodine for Korean adults as specified in the 2020 Dietary Reference Intakes for Koreans (KDRI)^(64)^. Further, 800 out of 116 827 participants were found to exceed the tolerable upper intake level (UL) of the 2020 KDRI for iodine^(64)^. The UL for adults in the 2020 KDRI was established by calculating the lowest observed adverse effect level and applying the uncertainty factor with reference to the average urine iodine output (2880 µg/l) of 17 men and women with simple goiter from a 2000 study that measured average urine iodine output according to dietary iodine intake in 184 patients with various thyroid diseases^(64,65)^. However, data on the correlation between dietary iodine intake and goiter in healthy Asians, who consume a variety of seaweed, are limited^(65)^. In previous studies, long-term consumption of a high amount of iodine (up to 2 g/d) did not lead to clinical adverse effects^(66,67)^. This because, in healthy individuals, the thyroid gland escapes the Wolff-Chaikoff effect, an autoregulatory mechanism and receives a consistent amount of iodine to maintain normal thyroid functions^(68)^. Further, the Korea Disease Control and Prevention Agency states that the normal range of thyroid stimulating hormone concentration in Koreans is 0·62–6·86 mmg/l, higher than that of Americans (0·45–4·12 mmg/l)^(69,70)^. In other words, the level of thyroid hormones and iodine metabolism in Koreans may differ from that among Westerners, as Koreans traditionally consume a large amount of seaweed^(69,70)^. A recent study hinted at the possibility of a racial gap in the genotypes of some iodine metabolism-related genes, such as thyroid peroxidase, Na-iodine symporter and thyroglobulin^(71)^. Such discrepancy is speculated to have contributed to the similar or lower prevalence of hypothyroidism and hyperthyroidism in Korea and Japan – two countries with high iodine intake – than that of other western countries^(72–74)^. A recent Korean study on premenopausal women reported a significantly reduced risk for metabolic syndrome among those with higher dietary iodine and seaweed intake^(20)^, calling for a re-evaluation of the scientific evidence underlying the recommended dietary allowance and UL of iodine intake for Koreans.

This study has a few limitations. First, though confounders were adjusted in a step-by-step approach when analysing the association between seaweed intake and T2DM risk by reviewing the literature and conducting preliminary analysis, there is still a possibility of residual confounders. Second, the KoGES database does not present data on intake of seaweed other than ‘laver’ and ‘kelp/sea mustard’, so it is possible that the seaweed intake of our participants may be underestimated. However, as of 2019, the daily seaweed intake of Koreans was about 75·56 g, and kelp (22·76 g), sea mustard (22·76 g) and laver (17·00 g) account for a significant proportion of their diet^(75)^. Hence, we believe that the data are a relatively good reflection of the actual level of seaweed intake^(75)^. Third, diet-related information was collected using a self-report questionnaire, so there is a possibility that a measurement error occurred. Despite the validation of the SQFFQ used in our study^(28)^, it is important to note that the validation process did not encompass validation for all individual food items, including seaweed intake. We acknowledge the significance of offering precise validation metrics tailored to seaweed intake, considering its unique dietary characteristics. Future research endeavours will benefit from addressing this particular validation aspect to ensure a comprehensive assessment of dietary measurements. Lastly, with regard to the use of self-reported T2DM diagnosis information, it is noteworthy that the information provided on the self-reported questionnaire was subject to validation through an individual interview conducted by a trained interviewer. This meticulous validation process serves to mitigate potential measurement errors, thereby enhancing the reliability of our gathered data.

Despite these limitations, this study has significant implications as the first study to prospectively analyse the association between intake of seaweed – a frequently consumed food in Korea – and T2DM risk.

### Conclusion

This study prospectively analysed the association between seaweed intake and T2DM risk in Korean adults aged 40 years or older who participated in the KoGES-HEXA study. The results indicated a dose-dependent inverse association between seaweed intake and T2DM risk among participants with normal weight. On the other hand, such an association was not observed in the participants with obesity. Amid limited study data on the prospective association between seaweed intake and T2DM risk in Korean adults, who consume a relatively large amount of seaweed compared to people in other countries, this study presents useful foundational data for T2DM management. Moreover, future research should prioritise considering major nutrients in seaweed such as iodine, fibre and carbohydrates that aim to prevent T2DM.
